# Micro- and Nanoplastics in the Environment: Analytical Approaches, Environmental Fate, Life Cycle, and Remediation Strategies—A Scoping Review

**DOI:** 10.3390/molecules31162789

**Published:** 2026-08-11

**Authors:** Dominika Kusyk, Beata Mruk, Ilona Górna, Magdalena Kowalówka, Izabela Bolesławska, Hanna Markowska, Sławomira Drzymała-Czyż

**Affiliations:** 1 Poznan University of Medical Sciences, Department of Bromatology, Rokietnicka 3, 60-806 Poznan, Poland; 87786@student.ump.edu.pl (D.K.); 87785@student.ump.edu.pl (B.M.); igorna@ump.edu.pl (I.G.); mkowalowka@ump.edu.pl (M.K.); ibolesla@ump.edu.pl (I.B.); hmarkowska8@gmail.com (H.M.); 2 Complex of Healthcare Institution, Kościuszki 96, 64-700 Czarnków, Poland

**Keywords:** microplastics, nanoplastics, analytical methods, environmental fate, life cycle assessment, remediation technologies, environmental pollution

## Abstract

Microplastics have emerged as one of the most widespread and significant environmental pollutants, occurring in aquatic and terrestrial ecosystems, the atmosphere, food, and living organisms. Given the rapid expansion of research in this field, a comprehensive synthesis of the current state of knowledge is warranted. The aim of this study was to map the available literature on microplastics, with particular emphasis on advanced identification and characterisation techniques, environmental transport and transformation processes, life cycle assessment, and remediation strategies. This scoping review was conducted in accordance with the PRISMA-ScR guidelines using publications retrieved from the PubMed, Scopus, Web of Science, and Google Scholar databases. The analysis demonstrated substantial advances in analytical methodologies, particularly spectroscopic and microscopic techniques, enabling the accurate characterisation of micro- and nanoplastic particles. It also highlighted the complex mechanisms governing the transport and transformation of microplastics across environmental compartments, as well as their important role as vectors of chemical contaminants. From a systems perspective, life cycle assessment of plastics was identified as an essential tool for evaluating their environmental impacts. Current mitigation approaches, including filtration technologies, wastewater treatment processes, and biological remediation strategies, were also reviewed, along with their limitations and potential for future development. This review identifies the lack of standardised analytical methodologies and the limited capability for nanoplastics detection as two major challenges hindering the global harmonisation of microplastics research. The findings emphasise the need for further interdisciplinary studies and the implementation of integrated strategies encompassing the entire life cycle of plastics to effectively reduce microplastics emissions and mitigate their environmental impacts.

## 1. Introduction

Plastic pollution is currently recognised as one of the most pressing environmental challenges of the twenty-first century [1]. Since the beginning of large-scale production of synthetic polymers in the mid-twentieth century, the global use of plastics has increased dramatically. Owing to their favourable physicochemical properties, including durability, resistance to environmental degradation, and cost-effective production, plastics have become indispensable materials across numerous industrial sectors [2]. However, these same characteristics have also contributed to the accumulation of vast quantities of plastic waste, the effective management of which remains a major environmental concern worldwide [3].

According to Plastics Europe, global plastics production reached approximately 430.9 million tonnes in 2024, reflecting the continued increase in plastic consumption worldwide and the growing importance of addressing plastic waste generation [4]. A substantial proportion of these materials is intended for short-term applications, particularly packaging. Following disposal, plastic waste frequently enters the environment, where it undergoes gradual degradation [5]. Exposure to ultraviolet radiation, oxidation, and mechanical abrasion results in the fragmentation of larger plastic items into microplastics [5]. Microplastics have now been detected in virtually every environmental compartment, including surface waters, soils, sediments, and the atmosphere [6,7]. Of particular concern is their occurrence in remote regions far from direct emission sources, such as the Arctic and high-altitude mountain environments, highlighting the importance of long-range atmospheric and oceanic transport processes [8].

Increasing attention has recently been paid to the presence of microplastics in food and drinking water intended for human consumption [9]. Microplastic particles have been identified in fish, seafood, table salt, honey, and drinking water [10]. Consequently, humans are continuously exposed to microplastics through both dietary intake and inhalation [11]. This growing exposure has attracted considerable interest in the fields of environmental toxicology and public health. Recent studies have reported the presence of microplastics in human blood, lungs, placenta, and breast milk [12,13,14,15]. Experimental evidence suggests that microplastics may induce oxidative stress, inflammatory responses, and disturbances of immune and metabolic functions [16,17]. Beyond their direct biological effects, microplastics can also act as carriers of environmental contaminants. Their surfaces readily adsorb persistent organic pollutants, pesticides, and heavy metals [18,19], thereby facilitating the transport and bioaccumulation of toxic substances in living organisms [20].

The increasing number of studies reporting microplastics in food, drinking water, and human tissues has intensified interest in reliable methods for their detection and quantitative determination [21]. These findings highlight the need for accurate and standardised analytical approaches to identify microplastics in environmental, food, and biological samples. In response, considerable progress has been made in developing advanced analytical techniques for microplastic characterisation. In particular, microscopic, spectroscopic, and spectrometric methods have become indispensable tools for determining the morphology, size, and chemical composition of microplastic particles [21]. In addition to analytical methodologies, increasing attention has recently been directed towards understanding the environmental fate of microplastics across their entire life cycle and the development of effective mitigation strategies. Therefore, this review not only summarises current analytical approaches but also highlights life cycle assessment (LCA) and remediation technologies as essential components for reducing microplastic pollution and supporting sustainable environmental management. Unlike many existing reviews focusing primarily on analytical techniques or environmental occurrence, this review places particular emphasis on the life cycle perspective of micro- and nanoplastics, integrating analytical methods, environmental fate, LCA, and remediation strategies into a single framework. This integrated perspective aims to identify critical stages of plastic emissions and highlight opportunities for more effective mitigation across the entire life cycle. Since the pioneering study by Thompson et al. [1], considerable progress has been made in understanding the sources, environmental transport, fate, analytical detection, and biological effects of microplastics, highlighting their significance as an emerging environmental and public health concern.

### 1.1. What Are Microplastics?

Microplastics are defined as synthetic polymer particles smaller than 5 mm in size [1,2]. According to the international standard ISO 24187:2023 [22], plastic debris is classified according to particle size into macroplastics (>25 mm), mesoplastics (5–25 mm), microplastics (1 μm–5 mm), and nanoplastics (<1 μm). Although alternative size classifications have been reported in the literature [3], the ISO terminology is increasingly adopted to improve consistency and comparability among environmental monitoring studies. Based on their origin, microplastics are categorised as either primary or secondary [23]. Primary microplastics are intentionally manufactured at microscopic dimensions for commercial applications, including cosmetic products, cleaning agents, and various industrial applications [6]. In contrast, secondary microplastics originate from the fragmentation of larger plastic items due to environmental weathering, including ultraviolet (UV) radiation, mechanical abrasion, oxidative processes, and microbial activity [5]. Microplastics occur in a wide range of morphological forms, including fibres, fragments, pellets, beads, films, and foams [7]. Their shape and size strongly influence their environmental transport, adsorption capacity for contaminants, and potential biological toxicity [8].

### 1.2. Sources of Microplastics

Microplastics originate from a wide variety of sources, including both industrial activities and everyday human practices [9]. The primary sources include microbeads used in personal care products, industrial plastic pellets employed during polymer manufacturing, and synthetic fibres released during the laundering of polyester, nylon, or acrylic textiles [6,10]. It has been estimated that a single washing cycle may release hundreds of thousands of synthetic fibres into wastewater [11]. Tire wear is also a major source of microplastic pollution, as the abrasion of vehicle tyres continuously generates polymer particles containing various chemical additives and heavy metals [12]. Secondary microplastics are primarily formed through the degradation of plastic packaging, agricultural films, fishing nets, polyethylene terephthalate (PET) bottles, and other plastic waste [5,13]. This degradation process is particularly intense in marine environments, where solar radiation and wave action accelerate the fragmentation of polymeric materials [14].

### 1.3. Occurrence in the Environment and Food

Microplastics have been detected in virtually all environmental compartments [15]. Recent global assessments have demonstrated that microplastics occur throughout the Earth’s ecosystems, including marine and freshwater environments, soils, the atmosphere, polar regions, mountain ecosystems, and remote locations far from direct emission sources, highlighting their ubiquitous environmental distribution [24,25]. Their presence has been confirmed in oceans, seas, rivers, lakes, groundwater, and drinking water [16,17]. They have also been identified in agricultural soils, where they may originate from organic fertilisers, sewage sludge, and the degradation of plastic materials used in agricultural practices [18]. Furthermore, atmospheric transport enables microplastics to travel over long distances, leading to their detection even in remote regions such as the Arctic and high-altitude mountain environments [19,20]. Increasing evidence also indicates the widespread occurrence of microplastics in food products. They have been detected in fish, seafood, table salt, honey, beer, milk, and bottled water, among other products [21,26]. Food contamination may result not only from environmental exposure but also from processing, packaging, and transportation [27].

### 1.4. Potential Effects on Human Health

**Figure 1:**
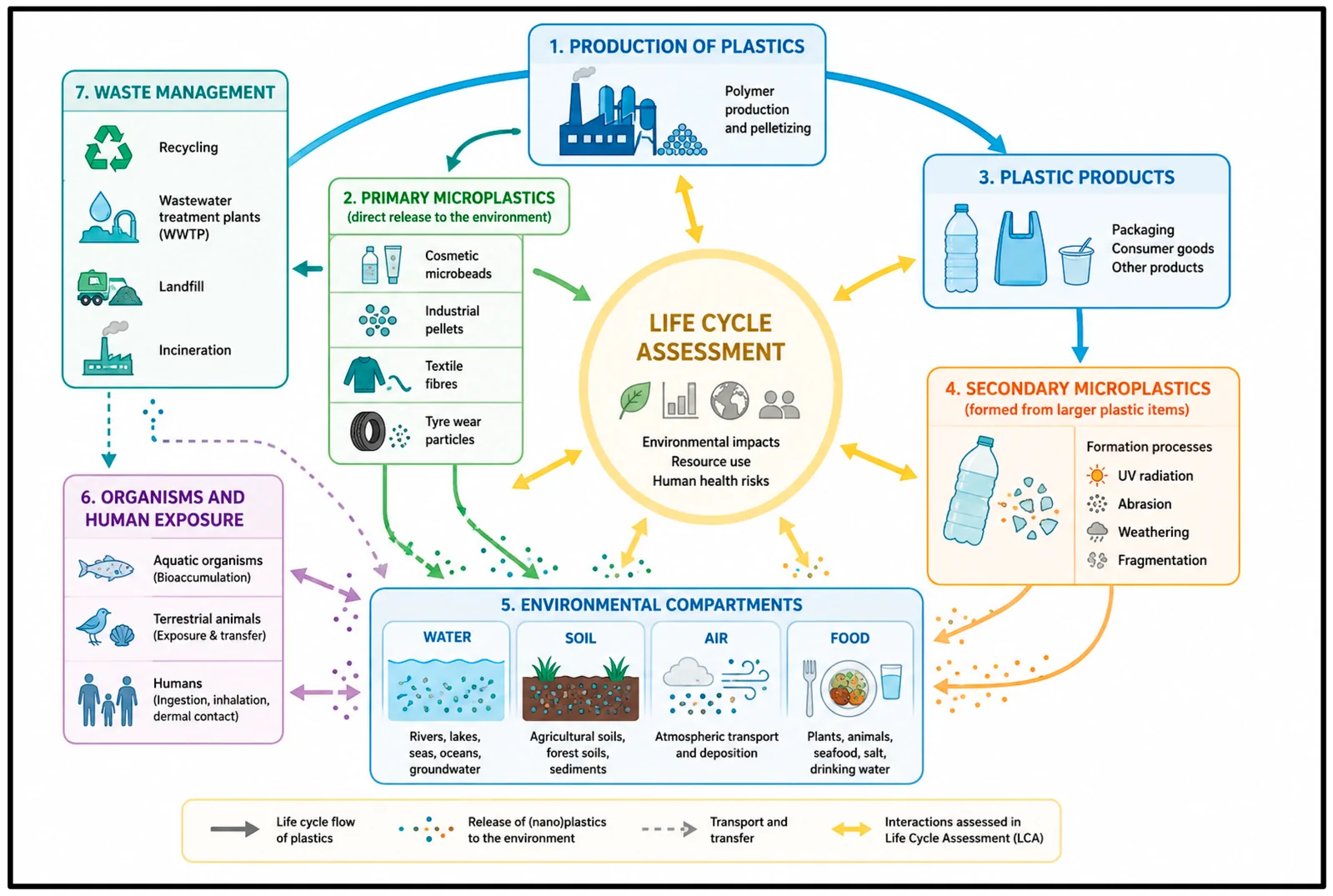
Life cycle and environmental pathway of microplastics.

The primary routes of human exposure to microplastics include the ingestion of contaminated food and drinking water, inhalation of airborne particles, and dermal contact [28]. In recent years, microplastics have been identified in human blood, lungs, placenta, breast milk, and gastrointestinal tissues [29,30,31,32]. These findings indicate that microplastics can cross biological barriers and translocate between organs [33]. However, particle size plays a decisive role in biological uptake. While larger microplastics are generally retained within the gastrointestinal tract and exhibit limited translocation, nanoplastics possess considerably greater potential to cross biological barriers, enter systemic circulation, accumulate in internal organs, and penetrate the placenta and blood–brain barrier. Both in vitro and in vivo studies suggest that microplastics may induce oxidative stress, inflammatory responses, and cellular damage [34]. Particular concern has been raised regarding their potential to disrupt endocrine, immune, and metabolic functions [34,35]. An additional health risk arises from microplastics’ ability to adsorb toxic chemicals, including heavy metals, pesticides, polychlorinated biphenyls (PCBs), and polycyclic aromatic hydrocarbons (PAHs) [36,37]. Consequently, microplastics may enhance the bioavailability of these contaminants to living organisms [38]. Despite the rapidly growing body of research, long-term epidemiological studies remain lacking, preventing a comprehensive assessment of the health consequences of chronic human exposure to microplastics [39]. A schematic representation of the microplastic life cycle is presented in Figure 1.

### 1.5. Aim of the Study

The aim of this review was to provide a comprehensive overview of current analytical approaches used for the identification and characterisation of microplastics in environmental, food, and biological samples. In addition, the review integrates current knowledge of analytical techniques, environmental behaviour, interactions with contaminants, life cycle assessment, and mitigation strategies to present a multidisciplinary perspective on micro- and nanoplastic pollution. Such an integrated approach may facilitate a better understanding of the challenges associated with the detection, environmental fate, and management of micro- and nanoplastics.

## 2. Micro- and Nanoplastics Characterisation Techniques

Accurate identification and characterisation of microplastics constitute fundamental components of contemporary environmental research. The considerable diversity in particle size, shape, colour, and chemical composition necessitates the use of advanced analytical techniques to ensure reliable analysis [40]. In recent years, substantial progress has been made in developing methods that enable both qualitative and quantitative assessment of microplastics in water, soil, sediments, food, and biological samples [41].

Methods used for microplastic characterisation can be broadly classified into four main groups: microscopic, spectroscopic, spectrometric, and advanced imaging techniques [42]. Each provides complementary information regarding the analysed particles. Microscopic methods enable the evaluation of particle morphology, size, and degree of degradation, whereas spectroscopic and spectrometric techniques facilitate polymer identification and the detection of additives incorporated during plastic manufacturing [43].

### 2.1. Microscopic Techniques

Microscopic techniques are among the most widely employed methods for the preliminary identification of microplastics. They enable the assessment of particle size, shape, colour, surface morphology, and degree of degradation [44]. These characteristics are essential for microplastic classification and for selecting appropriate analytical techniques for subsequent characterisation.

#### 2.1.1. Optical Microscopy

Optical microscopy is one of the oldest and most frequently applied techniques for microplastic analysis. It utilises visible light and optical lenses to generate magnified images of the particles under examination [45]. The principal advantages of optical microscopy include its simplicity, relatively low instrumentation costs, and the ability to examine large numbers of particles rapidly. The technique enables the determination of basic morphological characteristics, including particle length, width, shape, and colour [46].

Optical microscopy is particularly useful as a screening tool for environmental samples, enabling the differentiation of fibres, fragments, pellets, and films present in the material under analysis [47]. However, a major limitation of this technique is its inability to unequivocally identify the polymer composition. Particles with similar morphological characteristics may belong to different polymer types or even represent natural materials such as cellulose fibres [40]. Furthermore, the resolution of optical microscopy limits the detection of very small microplastic particles, generally below approximately 20 μm [48].

In routine laboratory practice, optical microscopy is frequently combined with the hot needle test, which enables rapid differentiation between plastic and non-plastic particles based on their thermal deformation. Although this method cannot identify polymer type, it provides a simple and inexpensive preliminary verification step before spectroscopic analysis [49].

#### 2.1.2. Scanning Electron Microscopy (SEM)

Scanning Electron Microscopy (SEM) is among the most advanced imaging techniques employed in microplastic research [50]. Unlike optical microscopy, SEM uses a focused electron beam to scan the sample surface. Interactions between electrons and the material generate signals used to produce high-resolution images [51].

SEM enables detailed examination of the surface morphology of microplastic particles, including cracks, pores, surface deformation, and signs of environmental weathering. These features provide valuable information regarding degradation processes induced by ultraviolet radiation, oxidation, and biological activity [52]. Owing to its high spatial resolution, SEM allows the analysis of particles measuring only a few micrometres or even smaller. Consequently, it is widely applied in environmental studies involving microplastics isolated from surface waters, sediments, and soils [53].

A major limitation of SEM is its inability to directly determine the chemical composition of polymers. Therefore, it is frequently combined with complementary analytical techniques, such as Raman spectroscopy or SEM coupled with energy-dispersive X-ray spectroscopy (SEM-EDX) [50].

#### 2.1.3. Scanning Electron Microscopy Coupled with Energy-Dispersive X-Ray Spectroscopy (SEM-EDX)

Scanning Electron Microscopy coupled with Energy-Dispersive X-ray Spectroscopy extends conventional SEM by integrating high-resolution imaging with elemental composition analysis [54]. During electron beam interaction with the sample surface, characteristic X-rays are emitted. Analysis of their energy enables the identification of the elements present on the surface of microplastic particles [55].

SEM-EDX facilitates the detection of additives commonly incorporated into plastics during manufacturing, including titanium-, calcium-, and silicon-containing compounds, as well as heavy metals. The technique is also valuable for assessing the adsorption of environmental contaminants onto microplastic surfaces [56]. Consequently, SEM-EDX is widely employed in studies investigating the environmental ageing of microplastics and their interactions with heavy metals and other environmental pollutants [54].

#### 2.1.4. Fourier Transform Infrared Spectroscopy (FTIR)

Fourier Transform Infrared Spectroscopy (FTIR) is currently one of the most widely applied techniques for polymer identification in microplastic research because it provides reliable chemical characterisation without destroying the analysed particles. The method identifies polymers by measuring the absorption of infrared radiation corresponding to characteristic molecular vibrations, allowing differentiation between common plastic types such as polyethylene, polypropylene, polyethylene terephthalate and polystyrene. Recent technological developments have substantially improved the analytical performance of FTIR. For example, large-area attenuated total reflectance FTIR (LAATR-FTIR) enables the detection of particles as small as approximately 1.3 μm, considerably extending the analytical capabilities of conventional FTIR systems for the investigation of smaller microplastics [57].

#### 2.1.5. Micro-FTIR and Raman Spectroscopy

Micro-FTIR enables automated identification of large numbers of particles while simultaneously providing information on their morphology and chemical composition. Recent studies have also demonstrated that synthetic FTIR spectral libraries may improve polymer identification when reference databases are incomplete [58]. Raman spectroscopy complements FTIR owing to its higher spatial resolution and ability to identify very small microplastic particles, while recent advances such as Surface-Enhanced Raman Spectroscopy (SERS) have further increased analytical sensitivity [59].

#### 2.1.6. Py-GC/MS and TED-GC/MS

Pyrolysis-gas chromatography/mass spectrometry (Py-GC/MS) and thermal extraction desorption gas chromatography/mass spectrometry (TED-GC/MS) are destructive techniques that provide highly sensitive quantitative information on polymer composition. Their application has recently expanded to human biomonitoring studies, including the detection of microplastics in human arteries [60] and in human semen and testicular tissue [61].

Table 1 provides a comparative overview of the principal analytical techniques used to identify and characterise microplastics and nanoplastics. The methods were evaluated with respect to particle size range, polymer identification capability, morphological analysis, quantification potential, suitability for nanoplastics, degree of destructiveness, automation potential, cost, and limits of detection.

## 3. Development of Analytical Methods for Micro- and Nanoplastics

The development of reliable analytical methods is one of the most critical aspects of microplastic research. Owing to the considerable diversity in particle size, morphology, and physicochemical properties, standardised procedures for sampling, isolation, identification, and quantitative determination are essential for obtaining accurate and reproducible results [62]. Particular attention must be paid to minimising the risk of secondary contamination and preventing sample loss during preparation and analysis [63].

### 3.1. Sampling

Sampling represents the first and one of the most crucial stages of microplastic analysis. Errors introduced at this stage may compromise the reliability and representativeness of the results [40].

Recent research has increasingly focused on the harmonisation of sampling protocols and quality assurance procedures to improve the comparability and reproducibility of microplastic studies across different environmental matrices. Standardised methodologies, together with rigorous contamination-control measures and detailed reporting of sampling conditions, are considered essential for reducing methodological variability and enabling reliable inter-study comparisons. In addition, the growing interest in nanoplastics has highlighted the need to adapt sampling strategies to ensure the recovery of increasingly smaller particles while minimising sample contamination and particle loss throughout the analytical workflow [64,65,66].

#### 3.1.1. Water

Surface water samples are commonly collected using plankton nets, filtration pumps, or automated sampling systems [67]. In marine studies, Manta trawl nets are widely employed to collect floating microplastic particles from the water surface [68]. Increasingly, large-volume filtration techniques are being applied to improve the detection of particles smaller than 300 μm [47].

#### 3.1.2. Soil

Microplastic analysis in soils is particularly challenging due to the complexity of the soil matrix. Soil samples are generally collected from predefined depths using stainless steel corers or soil samplers [69]. The use of plastic sampling equipment should be avoided to minimise the risk of secondary contamination [18].

#### 3.1.3. Food

Microplastic investigations in food encompass both plant- and animal-derived products. Microplastic particles have been detected in fish, seafood, table salt, drinking water, milk, and processed foods [26]. Sampling protocols must account for the possibility of contamination during transportation, storage, and laboratory processing [70].

#### 3.1.4. Biological Samples

Interest in the analysis of microplastics in human tissues and body fluids has increased substantially in recent years. Current studies include blood, breast milk, placenta, lung tissue, and faecal samples [71]. Because microplastics are generally present at very low concentrations in biological matrices, rigorous contamination-control procedures are essential throughout sample collection and processing [72].

### 3.2. Isolation and Extraction of Microplastics

Following sample collection, microplastic particles must be separated from the surrounding environmental or biological matrix. Various extraction and purification techniques have been developed for this purpose [47]. Although the NOAA Guidelines constitute an important methodological foundation for the isolation and extraction of microplastics, they should not be considered an exhaustive overview of currently available analytical approaches. Recent comprehensive reviews and book chapters have expanded the methodological framework by discussing extraction protocols for a wide range of environmental and biological matrices, including water, sediments, soils, sludge, wastewater, compost, and biota. These publications also evaluate sample drying, sieving, organic matter removal, density separation, filtration, purification procedures, recovery tests, contamination control, and matrix-specific recommendations for selecting appropriate extraction strategies [73,74,75].

#### 3.2.1. Filtration

Filtration is among the most widely used methods for microplastic isolation. The sample is passed through filters with defined pore sizes that retain microplastic particles while allowing smaller components to pass through [45]. Depending on the analytical objective, glass fibre, quartz, membrane, or metal filters may be employed [36].

#### 3.2.2. Density Separation

Density separation exploits the density difference between microplastics and the sample’s mineral components [76]. Saturated solutions of sodium chloride (NaCl), zinc chloride (ZnCl_2_), or sodium iodide (NaI) are commonly used [77]. Because most plastic polymers are less dense than these solutions, they float to the surface while heavier mineral particles settle at the bottom [78].

#### 3.2.3. Chemical Digestion

Chemical digestion is primarily applied to biological and food samples to remove organic matter without significantly damaging microplastic particles [79]. Commonly used reagents include hydrogen peroxide (H_2_O_2_), potassium hydroxide (KOH), and proteolytic enzymes [80].

### 3.3. Identification and Quantification

Following isolation, the number, size, and polymer composition of microplastic particles must be determined [81]. This is typically achieved using the analytical techniques described in Section 3, particularly Fourier Transform Infrared Spectroscopy (FTIR), micro-FTIR, Raman spectroscopy, and pyrolysis–gas chromatography–mass spectrometry (Py-GC-MS) [82]. Qualitative analysis enables polymer identification, whereas quantitative analysis determines particle abundance per unit volume, mass, or surface area [43]. Increasingly, automated image analysis systems supported by artificial intelligence algorithms are being implemented to improve the speed, reproducibility, and accuracy of microplastic detection and quantification [83]. Recent advances have also introduced complementary analytical techniques, including near-infrared spectroscopy (NIR), nuclear magnetic resonance (NMR), and X-ray fluorescence (XRF), for microplastic identification and characterisation. Although these methods are less frequently employed than FTIR or Raman spectroscopy, they provide valuable information on polymer composition, additive content, and elemental characteristics, thereby expanding the analytical toolbox available for comprehensive microplastic analysis [84,85,86,87].

### 3.4. Analytical Challenges

#### 3.4.1. Sample Contamination

One of the most common analytical challenges is secondary contamination arising from airborne microplastics, laboratory clothing, or analytical equipment [88]. Synthetic textile fibres released from laboratory garments are considered a major source of contamination [89].

#### 3.4.2. Sample Loss

Microplastic particles may be lost during filtration, density separation, or chemical digestion procedures [90]. This issue is particularly significant for the smallest particles, which may pass through filters or become damaged during sample preparation [91].

#### 3.4.3. Lack of Method Standardisation

Despite rapid advances in microplastic research, universally accepted protocols for sampling, extraction, and particle identification remain unavailable [92]. This lack of methodological standardisation limits the comparability of results among studies and hinders comprehensive global assessments of microplastic contamination [93].

## 4. Interactions Between Micro- and Nanoplastics and Environmental Contaminants

Microplastics are not merely passive environmental pollutants. Owing to their physicochemical properties, they interact with numerous chemical contaminants, influencing their transport, bioavailability, and bioaccumulation within ecosystems [38].

### 4.1. Sorption of Organic Contaminants

Microplastic surfaces possess a high adsorption capacity. Persistent organic pollutants (POPs), including polychlorinated biphenyls (PCBs), organochlorine pesticides, and polycyclic aromatic hydrocarbons (PAHs), readily accumulate on their surfaces [36]. Sorption efficiency depends on polymer type, the degree of weathering, and the physicochemical properties of the contaminant [37]. Environmental ageing increases surface roughness and porosity, thereby enhancing the adsorption capacity of microplastic particles [52].

### 4.2. Desorption and Release of Contaminants

Following ingestion by living organisms, microplastics may release previously adsorbed chemical contaminants through desorption processes [94]. The rate of contaminant release depends on environmental conditions, including temperature, pH, and the presence of surfactants [95]. Desorption may increase the bioavailability of toxic compounds, thereby posing an additional ecological risk [96].

### 4.3. Microplastics as Vectors of Toxic Substances

Microplastics can serve as vectors transporting chemical contaminants between different environmental compartments [97]. In addition to adsorbed pollutants, plastic particles may contain additives incorporated during manufacturing, including bisphenol A (BPA), phthalates, UV stabilisers, and flame retardants [98]. These substances may gradually leach into the environment or biological tissues, increasing the potential for toxic effects [34].

### 4.4. Bioaccumulation

Microplastics are ingested by aquatic and terrestrial organisms across multiple trophic levels [99]. Their presence has been documented in zooplankton, molluscs, crustaceans, fish, birds, and marine mammals [100]. The transfer of microplastics through food webs may contribute to the bioaccumulation and biomagnification of associated contaminants [101].

### 4.5. Implications for Human Health and Ecosystems

Interactions between microplastics and chemical contaminants may substantially increase the exposure of living organisms to hazardous substances [33]. Experimental studies indicate that microplastics can exacerbate oxidative stress, inflammatory responses, and disturbances in endocrine and immune system function [35]. At the ecosystem level, microplastics may affect biodiversity, trophic interactions, and biogeochemical processes [102]. Consequently, understanding the mechanisms governing interactions between microplastics and environmental contaminants remains a major priority in contemporary environmental research.

## 5. Life Cycle Assessment of Micro- and Nanoplastics

### 5.1. Plastic Production

LCA is one of the most valuable tools for identifying solutions with the lowest potential environmental impact. It is a standardised analytical methodology for evaluating the environmental impacts of products, processes, and technologies throughout their life cycle. Furthermore, LCA supports the assessment and improvement of environmental sustainability by linking emissions and environmental burdens with their potential impacts on ecosystems [103].

In microplastic research, LCA provides a systems-based framework for evaluating plastic emissions throughout the life cycle of plastic products. However, current LCA models still incorporate plastic emissions only to a limited extent during the Life Cycle Impact Assessment (LCIA) phase. In many cases, plastic emissions are treated merely as material flows without adequately characterising their environmental consequences, potentially leading to an underestimation of their actual environmental impact [104]. This limitation hampers the evaluation of strategies to reduce microplastic emissions [105].

The life cycle of plastics comprises three principal stages: production, use, and end-of-life management. During the production phase, fossil resources such as crude oil, natural gas, and coal are converted into polymeric materials through energy-intensive industrial processes [105]. Global plastic production exceeded 368 million tonnes annually in 2019, largely driven by the growing demand for single-use products. Increased production has led to the accumulation of plastic waste and the continuous release of micro- and nanoplastics throughout the plastic life cycle. Consequently, living organisms are exposed not only to plastic particles but also to the chemical additives associated with them. Nevertheless, significant knowledge gaps remain regarding the full extent of these environmental and biological impacts [106].

Modern plastic production is dominated by polyethylene (PE), polypropylene (PP), polystyrene (PS), polyethylene terephthalate (PET), and polyvinyl chloride (PVC). These polymers are extensively used in packaging, consumer products, and industrial applications. During use and following disposal, they undergo gradual weathering and fragmentation, generating micro- and nanoplastics. Despite their environmental significance, these emissions are not yet fully incorporated into LCIA models, representing a major challenge for the holistic life cycle assessment of plastic materials [107].

### 5.2. Use Phase

The use phase of plastic products represents one of the major sources of microplastic emissions into the environment [108]. In the case of synthetic textiles, the primary source of emissions is microfibers (MFs) released during the washing of fabrics such as polyester (PES) and polyamide (PA). Natural fibres containing technological additives introduced during textile manufacturing may also be released. These microfibers enter the environment either directly through wastewater or indirectly via wastewater treatment plants, which do not always remove them efficiently [109].

Transportation systems also constitute a significant source of microplastic pollution. During tyre wear, synthetic rubber particles are generated, forming so-called tyre-and-road wear particles (TRWPs). These emissions are particularly high in areas with heavy traffic and represent an important source of environmental microplastic contamination [110].

Plastic products also degrade due to ultraviolet (UV) radiation, oxygen exposure, and changing weather conditions. These processes weaken the polymer structure, leading to fragmentation and the release of small plastic particles into the environment. Mechanical activities such as abrasion, drilling, and sanding further accelerate microplastic formation. Polymer ageing is additionally promoted by the migration of additives, including plasticisers, from the interior of the material to its surface. This process reduces polymer flexibility, increases brittleness, and enhances susceptibility to degradation and fragmentation into microplastics [111].

### 5.3. Environmental Release

Once released into the environment, microplastics undergo transport, fragmentation, and accumulation across various ecosystem compartments. Agricultural mulch films represent an important source of soil contamination. These materials, typically composed of PE or PVC, gradually degrade into microplastics under the influence of UV radiation, weathering, and mechanical stress. Agricultural practices such as ploughing further promote fragmentation and the incorporation of microplastic particles into soil [112].

Microplastics originating from agricultural films may also contain numerous chemical additives, including plasticisers, stabilisers, and UV absorbers. These compounds can be released into the soil environment, altering its biological and physicochemical properties. Consequently, such contamination poses a long-term threat to agroecosystem quality [112].

In aquatic environments, the behaviour of microplastics depends on their density, size, shape, and polymer composition. Particles may remain suspended, float on the water surface, or settle into sediments. Processes such as biofouling and the adsorption of organic matter further modify particle density and transport pathways. Water distribution systems and drinking water treatment facilities also contribute to microplastic contamination through the degradation of pipe materials and the deterioration of membrane filters. Although water treatment technologies achieve high removal efficiencies, they do not completely eliminate microplastics, allowing their continued transport within aquatic systems [113].

### 5.4. Recycling and Waste Management

At the end of their service life, plastic materials may undergo mechanical recycling, chemical recycling, energy recovery through incineration, or landfill disposal. Although these approaches reduce the amount of plastic waste entering the environment, each is associated with specific environmental consequences, including the potential for secondary microplastic emissions [114].

Mechanical recycling is currently the most widely used approach and involves sorting, shredding, washing, and reprocessing plastic waste. While this process reduces the demand for virgin raw materials, intensive mechanical treatment may generate considerable amounts of microplastic particles. Such particles have been detected in both process wastewater and sewage sludge produced during wastewater treatment [115].

Chemical recycling represents an alternative strategy in which polymers are depolymerised into monomers or other chemical feedstocks. This technology enables the processing of more complex waste streams and may reduce the direct generation of microplastics. However, it is highly energy-intensive, and its environmental performance should therefore be evaluated from a life cycle perspective [116].

For plastic waste unsuitable for material recovery, incineration with energy recovery is commonly applied. This method reduces waste volume and limits further environmental fragmentation; however, it may contribute to greenhouse gas emissions and the release of other atmospheric pollutants [115].

A substantial proportion of plastic waste is still disposed of in landfills [115]. Under landfill conditions, plastics gradually degrade due to UV radiation and fluctuations in temperature and moisture, resulting in the formation of micro- and nanoplastics. These particles may subsequently migrate into soil, groundwater, and the atmosphere [117].

Although recycling is an essential strategy for mitigating plastic pollution, the recycling process itself may generate secondary microplastic emissions. Therefore, increasing recycling rates should be accompanied by the development of efficient wastewater treatment technologies and emission control systems [118].

### 5.5. Environmental Impact

Microplastics pose a significant environmental threat due to their persistence, widespread occurrence, and interactions with living organisms [119]. They have been detected in water, soil, the atmosphere, and organisms occupying various trophic levels. Furthermore, microplastics can adsorb chemical contaminants and serve as carriers for microorganisms, thereby amplifying their environmental impacts [120].

Microplastic pollution is also closely linked to climate change. During the degradation of plastic materials, greenhouse gases such as carbon dioxide (CO_2_), methane (CH_4_), and nitrous oxide (N_2_O) may be released. The plastics sector is estimated to account for approximately 3.3% of global greenhouse gas emissions. Conversely, climate change may alter the degradation rate and environmental transport of microplastics, further strengthening the interactions between these two global challenges [121].

Another major concern is the substantial consumption of natural resources throughout the plastic life cycle. Plastic production requires considerable amounts of fossil fuels, energy, and water, while increasing volumes of plastic waste place additional pressure on ecosystems. The limited efficiency of current waste management systems allows a substantial fraction of plastic waste to enter the environment directly, incurring additional environmental costs for cleanup and remediation [122].

## 6. Strategies for Mitigating Micro- and Nanoplastic Pollution

Mitigating microplastic pollution requires implementing strategies that address both the removal of particles already present in the environment and the prevention of emissions at the source [118]. Numerous approaches have been developed in recent years, differing in their mechanisms of action, efficiency, and technological readiness. The principal strategies are summarised in Table 2.

### 6.1. Filtration Technologies

Filtration is one of the most important approaches for reducing microplastic emissions into aquatic environments. Owing to the widespread occurrence of these contaminants in municipal wastewater, industrial effluents, and surface waters, the development of efficient filtration technologies represents a key component of microplastic mitigation strategies. Conventional approaches such as sand filtration are currently used alongside advanced membrane technologies and next-generation adsorbent materials [123].

Rapid sand filtration is among the most commonly applied techniques. It offers high hydraulic capacity, relatively low space requirements, and limited sensitivity to water-quality fluctuations. Studies have demonstrated that fine sand fractions (0.5–1.0 mm) can remove approximately 90% of larger fibrous microplastics. However, its efficiency decreases substantially for particles smaller than 200 μm, making additional treatment steps necessary [123,124].

Biochar-based filtration has emerged as a promising alternative. Biochar filters can remove more than 95% of 10 μm microplastic microbeads. Their high performance results from the biochar’s highly porous structure, which efficiently traps and immobilises particles within the filter matrix. Consequently, biochar represents a relatively inexpensive option for improving the efficiency of existing water treatment systems [125].

Membrane technologies are increasingly recognised as one of the most effective methods for microplastic removal. Microfiltration membranes can retain more than 99% of particles present in wastewater by acting as a physical barrier that prevents their passage into treated water. Despite higher investment and operational costs, these technologies are regarded as essential components of future wastewater treatment systems [126].

Despite the high efficiency of currently available filtration technologies, none provides complete removal of all microplastic fractions. The smallest particles remain particularly challenging to eliminate because they may pass through conventional filtration systems. Consequently, ongoing research focuses on optimising operational parameters and developing novel filtration materials with enhanced removal efficiency [127].

### 6.2. Wastewater Treatment

Wastewater treatment plants (WWTPs) play a crucial role in limiting the release of microplastics into aquatic environments. They represent one of the primary barriers preventing these contaminants from reaching rivers, lakes, and marine ecosystems. Mechanical, biological, and chemical treatment processes are employed to remove microplastic particles from wastewater [128].

**Table 2:** Effectiveness of microplastic mitigation strategies.

Strategy	Mechanism of Action	Effectiveness	Limitations	References
Sand filtration	Physical retention of microplastic particles	Up to ~90% removal of larger particles	Low efficiency for particles <200 μm	[123,124]
Biochar filters	Adsorption and entrapment of particles within the filter matrix	>95% removal of ~10 μm microbeads	Requires further validation	[125]
Membrane filtration	Physical barrier	>99% removal	High operational costs	[126]
Wastewater treatment	Mechanical and biological treatment	57–99% removal	Microplastics accumulate in sewage sludge	[129,130,131,132,133]
Bioremediation	Enzymatic degradation	Promising; efficiency depends on microorganism, polymer type, and environmental conditions; currently insufficient data for generalisation	Long treatment time; performance strongly influenced by temperature, pH, oxygen availability, and microbial community composition	[134,135,136,137,138,139]
Recycling and waste management	Prevention of emissions at the source	High environmental effectiveness	Dependent on waste management infrastructure and system organisation	[140,141,142,143,144,145]

Modern WWTPs can remove between 57% and 99% of microplastics. Particularly high removal efficiencies are achieved in facilities equipped with advanced secondary and tertiary treatment technologies [129]. For example, two wastewater treatment plants in Chennai, India, achieved microplastic removal efficiencies of approximately 96% and 93%, respectively. Despite these high removal rates, substantial numbers of particles continue to be discharged with treated effluent because of the enormous volumes of wastewater processed daily [130].

Most retained microplastics are not completely eliminated but instead accumulate in sewage sludge. A major concern is their potential reintroduction into the treatment process through sidestreams generated during sludge processing. It has been estimated that approximately 12% of the microplastics accumulated in sewage sludge may re-enter the treatment system, particularly polyester microfibers [131].

Subsequent sludge treatment processes also influence the fate of microplastics. Anaerobic digestion has been shown to reduce the number of microplastic particles, whereas certain stabilisation methods, such as lime treatment, may promote the fragmentation of larger particles into smaller ones [132]. Hydrothermal treatment technologies have also attracted increasing attention because they can modify the chemical properties of microplastics and reduce their environmental impacts [133].

Despite the high efficiency of wastewater treatment plants, microplastic pollution has not been completely eliminated. Microplastics retained in sewage sludge may ultimately enter the environment when sludge is applied to agricultural land. Therefore, continued technological improvements in wastewater treatment, together with measures to reduce plastic emissions at the production and consumption stages, remain essential for effective control of microplastic pollution [129].

### 6.3. Bioremediation

Bioremediation involves the removal of contaminants using living organisms, primarily microorganisms that degrade or transform harmful substances. In the context of microplastic pollution, this approach represents a promising alternative to physical and chemical methods, which often separate contaminants without achieving their complete degradation [134].

Bacteria and fungi play a central role in bioremediation, as they can adapt to the presence of plastic materials in the environment. In response to microplastic-induced stress, these microorganisms increase their metabolic activity and produce enzymes involved in polymer degradation [135]. Certain algae have also been shown to contribute to degradation processes, particularly in aquatic environments [136].

The mechanism of bioremediation is primarily based on enzymatic biodegradation. Microorganisms colonise plastic surfaces, forming biofilms that facilitate enzyme contact with polymeric materials [137,138]. These enzymes break long polymer chains into smaller compounds and monomers, which are subsequently utilised as sources of carbon and energy. Particular attention has been given to the enzymes PETase and MHETase for their ability to degrade polyethylene terephthalate (PET) [134].

The principal advantage of bioremediation lies in its ability to achieve the actual degradation of polymers into simpler and less harmful compounds. Unlike many physical treatment methods, bioremediation has the potential to result in the complete mineralisation of microplastics [135].

However, this technology also has several limitations. Biodegradation is generally a slow process, and its efficiency depends strongly on environmental factors such as temperature, pH, oxygen availability, and the composition of microbial communities. Furthermore, the number of known microorganisms and enzymes capable of efficiently degrading plastics remains limited, and most of the available evidence comes from laboratory-scale studies [134].

Currently, bioremediation is regarded as one of the most promising approaches for mitigating microplastic pollution. Nevertheless, its practical application requires further investigation into biodegradation mechanisms and the feasibility of implementing these processes on an industrial scale [139].

### 6.4. Waste Management

Waste management constitutes one of the key components of strategies aimed at reducing environmental microplastic pollution. The rapid increase in global plastic production—from approximately 2 million tonnes in 1950 to 460 million tonnes annually in 2019-has resulted in a substantial rise in plastic waste generation and microplastic emissions into the environment [140].

Only a small proportion of plastic waste is currently recycled, whereas a considerable amount is disposed of in landfills or released directly into the environment. Consequently, increasing attention has been directed toward strategies that reduce waste generation and improve the efficiency of waste management systems [141].

The most effective strategy for reducing microplastic emissions is to prevent the generation of plastic waste at the source [142]. This approach aligns with the principles of the circular economy, which aims to reduce the consumption of virgin resources, extend product lifespans, and maximise material reuse. Preventive measures are particularly important because plastics are highly persistent and tend to fragment into progressively smaller particles rather than undergoing complete degradation [143].

Plastic recycling is another essential component of sustainable waste management. Mechanical recycling involves sorting, shredding, washing, and reprocessing polymeric materials, thereby reducing landfill disposal and the consumption of virgin raw materials. However, these processes may also generate additional microplastic particles released into the air, soil, and surface waters [118]. Chemical recycling is an alternative approach that breaks polymers down into simpler chemical compounds that can be used as feedstocks for manufacturing new materials. Although this method enables the recovery of plastics that are unsuitable for mechanical recycling, it still requires further technological development and a comprehensive environmental assessment [144].

Selective waste collection also plays a crucial role by improving material recovery and increasing recycling efficiency. The absence of effective waste segregation systems remains one of the major reasons for low recycling rates, particularly in developing countries. Plastic waste that escapes formal waste management systems gradually degrades, becoming an important source of secondary microplastics [140].

Landfills may also act as significant sources of microplastic pollution [142]. Prolonged exposure to UV radiation, moisture, and temperature fluctuations promotes the fragmentation of discarded plastic materials. The resulting microplastic particles may subsequently be transported through landfill leachate or dispersed by wind into soils, surface waters, and groundwater [144]. Sewage sludge applied in agriculture may constitute an additional source of environmental microplastics [140].

Legislative measures are likewise essential for reducing microplastic emissions. Many countries have introduced bans on plastic microbeads in cosmetic products and restrictions on the intentional addition of microplastics to consumer products. Within the European Union, particular emphasis has been placed on reducing the use of single-use plastics and increasing recycling rates [140]. There is also a growing recognition of the need to establish harmonised microplastic monitoring systems and implement comprehensive waste management strategies that combine technological innovations with educational initiatives [145].

Overall, effective waste management plays a pivotal role in mitigating microplastic pollution. Although recycling and advanced waste treatment technologies can substantially reduce the scale of the problem, preventing plastic waste generation at its source remains the most effective long-term solution [145].

### 6.5. Future Directions for Mitigating Microplastic Pollution

Current projections indicate that, without effective intervention, environmental microplastic contamination will continue to increase. It is estimated that between 10 and 40 million tonnes of microplastics are released into the environment annually, and this amount may double by 2040 if current trends persist [1]. Continued growth in global plastic production is also anticipated [146], further contributing to the accumulation of microplastics in aquatic environments, soils, and living organisms [147].

One of the most promising research directions involves biodegradable plastics such as polylactic acid (PLA) and polyhydroxyalkanoates (PHA). These materials can be produced from renewable resources and biodegraded by microorganisms, making them potential alternatives to conventional petroleum-based plastics. However, recent studies indicate that their degradation may also generate small particles referred to as biomicroplastics. Therefore, further investigation into their long-term environmental impacts is required [148].

Reducing microplastic emissions during product use also represents an important strategy. Washing machine filters capable of capturing synthetic microfibers released during laundry have attracted particular attention. An example of implementing this approach is the French legislation requiring newly manufactured washing machines to be equipped with microfiber filtration systems [147].

Advanced sorbent materials for removing microplastics from water and wastewater have also emerged as a promising area of research. These materials include biochar, graphene, and various nanomaterials characterised by high specific surface areas and excellent adsorption capacities. Although these technologies have the potential to improve wastewater treatment and drinking water purification, their large-scale implementation requires further evaluation regarding efficiency, cost-effectiveness, and environmental safety [149,150].

Artificial intelligence (AI) is increasingly being applied to microplastic monitoring and analysis. Machine learning algorithms enable the automated detection and classification of particles based on microscopic images and spectroscopic data. These approaches may substantially accelerate analytical workflows, improve identification accuracy, and facilitate large-scale environmental monitoring. AI is also being used to support the development of novel biodegradation and remediation strategies [151].

Another promising research area involves advanced microplastic degradation technologies, including photocatalysis, advanced oxidation processes, and plasma-based treatments. These techniques promote the cleavage of chemical bonds in polymers, converting microplastics into simpler compounds. Particularly encouraging results have been obtained using photocatalytic and electrochemical processes, which may considerably accelerate plastic degradation compared with natural environmental processes [152].

Eco-design principles are also expected to play an increasingly important role. This approach incorporates environmental considerations into product design by selecting more durable materials, reducing surface abrasion, and minimising microplastic release during product use. Such strategies are fully consistent with the principles of the circular economy [147,152].

Nevertheless, effectively mitigating microplastic pollution requires not only technological innovation but also appropriate legislation, public education, and international cooperation. Experts emphasise the need for a life cycle approach encompassing plastic design, production, use, waste management, and environmental remediation. Only the integration of technological, legislative, and societal measures will effectively reduce microplastic pollution in the future [153,154].

## 7. Challenges and Future Research Directions

### 7.1. Lack of Methodological Standardisation

One of the greatest challenges in microplastic research is the absence of standardised methods for sampling, extraction, identification, and quantitative analysis. Current protocols differ considerably among laboratories with respect to sample preparation, filter mesh size, density separation procedures, and polymer identification techniques, making comparisons between studies difficult [155].

This issue is particularly evident in food-related studies, where highly variable isolation and identification protocols are employed [156]. Standardised analytical procedures are also lacking for sediments, biological tissues, and especially nanoplastics [157]. Therefore, the development of standardised operating procedures and validated sample-preparation methods is considered one of the highest priorities for future microplastic research [158].

### 7.2. Challenges in Data Interpretation

The interpretation of microplastic research findings is complicated by the remarkable diversity of particles in terms of size, shape, and chemical composition [156]. The smallest particle fractions are particularly problematic because they often go undetected due to limitations in current extraction and identification techniques [155].

Additional challenges arise from polymer ageing and the presence of organic and inorganic contaminants, which may interfere with spectroscopic analyses and hinder accurate polymer identification [159]. Considerable uncertainty also remains regarding the health consequences of human exposure to microplastics. Despite the rapidly expanding body of research, current evidence remains insufficient to permit a definitive assessment of the associated health risks [160].

### 7.3. Research Gaps

Despite substantial progress in recent years, significant knowledge gaps remain regarding the environmental fate of microplastics. In freshwater ecosystems, the processes of bioaccumulation, trophic transfer, and biomagnification have not yet been fully elucidated [161].

Similarly, limited information is available on the occurrence and distribution of microplastics in wetland ecosystems, which may function as both sinks and secondary sources of contamination [162]. Increasing attention is also being devoted to the atmospheric transport of microplastics and their role in the global environmental cycling [163]. However, one of the most critical research gaps concerns nanoplastics, for which reliable detection methods and comprehensive toxicological data remain insufficient [157].

### 7.4. Future Perspectives

One of the primary objectives of future research will be to establish globally accepted analytical standards that enable direct comparison of results generated by different research groups [155]. Equally important will be the development of more sensitive analytical techniques capable of detecting the smallest particles, particularly nanoplastics [157].

In atmospheric research, advanced spectroscopic methods and thermoanalytical techniques coupled with mass spectrometry are expected to play an increasingly important role [163]. Bibliometric analyses also indicate rapid growth in research focused on microplastic removal technologies based on constructed wetlands, biological treatment processes, and photocatalysis [164]. Furthermore, evaluating the long-term effects of microplastic accumulation in human tissues and organs will remain a major research priority [160].

**Figure 2:**
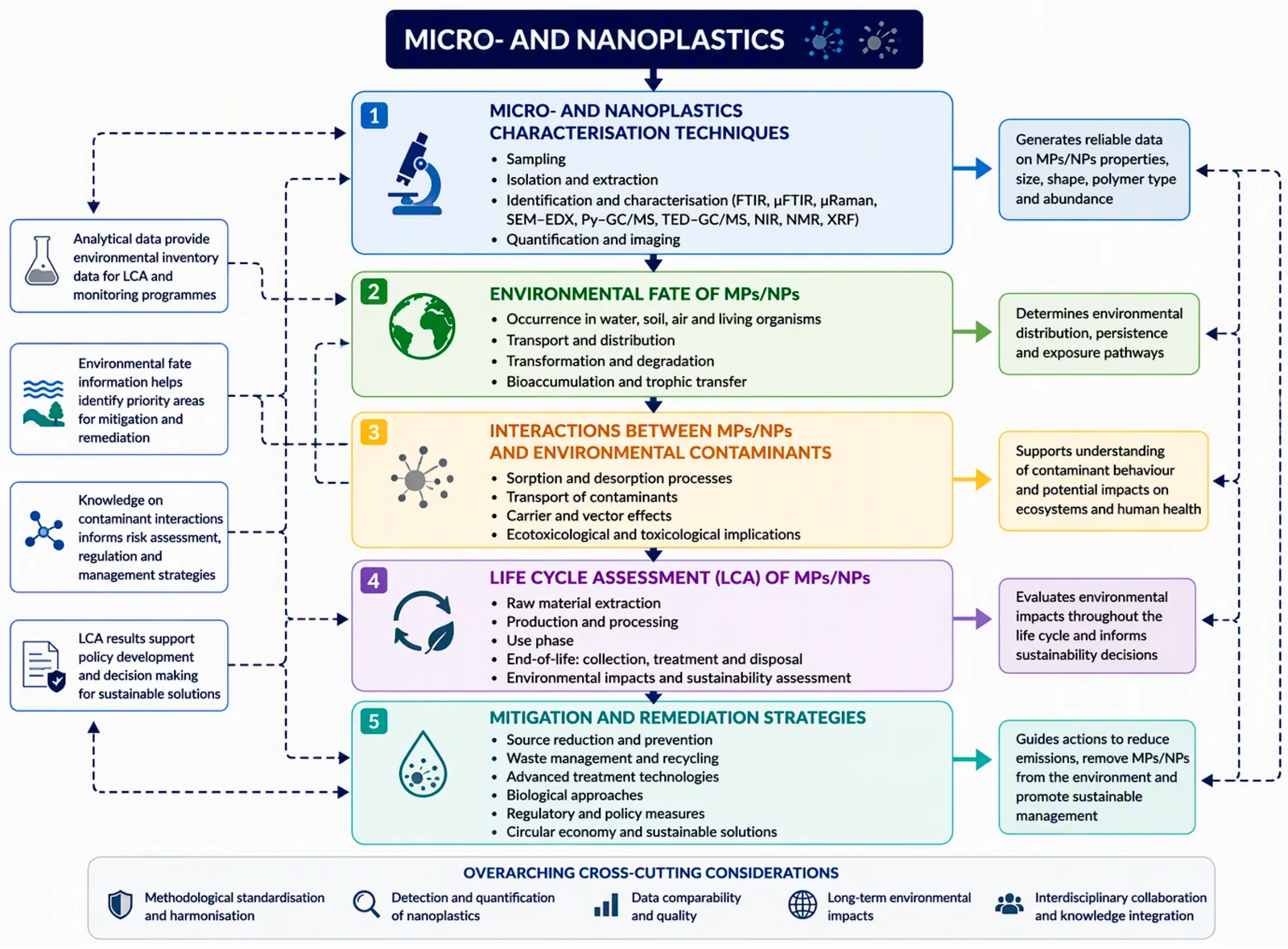
Integrated framework of micro- and nanoplastics characterisation, environmental fate, impacts, and mitigation strategies.

To provide a synthesis-oriented summary of the topics discussed in this review, Table 3 presents the current state of knowledge, key challenges, research gaps, and future research directions across the principal areas of micro- and nanoplastic research. The table integrates information related to analytical methods, environmental fate, interactions with environmental contaminants, life cycle assessment, and remediation strategies. Figure 2 complements this synthesis by illustrating the conceptual relationships among these research areas. The framework demonstrates that micro- and nanoplastic characterisation techniques constitute the foundation for studies investigating environmental fate, contaminant interactions, life cycle assessment, and mitigation and remediation strategies.

## 8. Materials and Methods

This study was conducted as a scoping review to comprehensively map and synthesise the current state of knowledge on microplastics. The review covered topics related to the identification and characterisation of microplastics; their occurrence, environmental transport, and transformation; potential impacts on human health and ecosystems; and current pollution mitigation strategies and remediation approaches.

**Figure 3:**
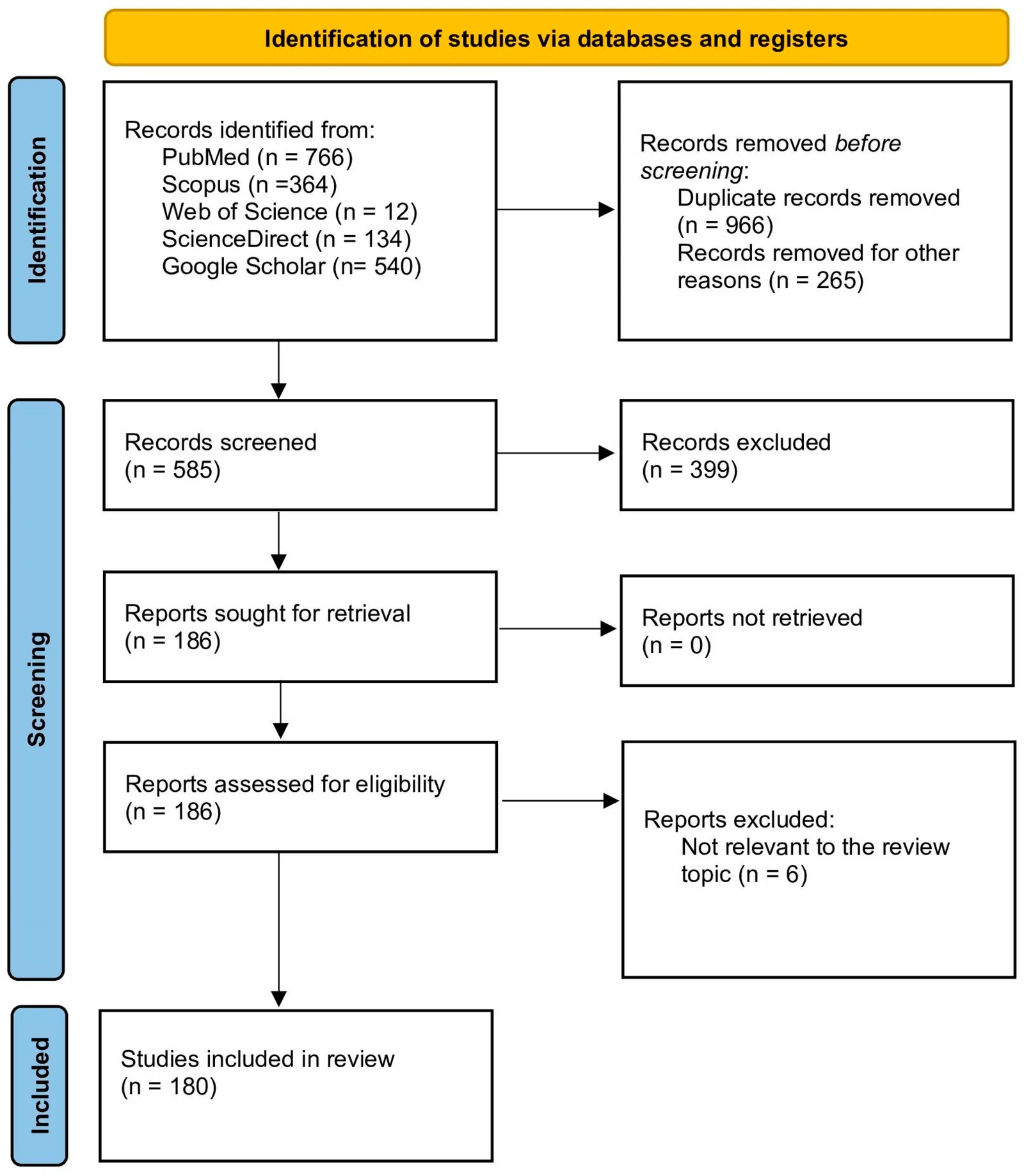
PRISMA-ScR flow diagram of the literature search, screening, eligibility assessment, and study selection process.

The literature search was performed in accordance with the PRISMA Extension for Scoping Reviews (PRISMA-ScR) guidelines (Figure 3). Publications were retrieved from the PubMed, Scopus, Web of Science, and Google Scholar databases using a predefined search strategy based on combinations of the following keywords: “microplastics,” “nanoplastics,” “analytical techniques,” “environmental distribution,” “ecotoxicology,” “life cycle assessment,” and “remediation.” Eligible studies included full-text articles published in English, comprising both original research papers and review articles addressing the identification, characterisation, environmental transport, environmental and health impacts of microplastics, as well as methods for their removal. Conference abstracts, editorials, letters to the editor, non-full-text publications, and studies not directly related to the scope of this review were excluded. The last literature search was conducted on 26 June 2026.

Google Scholar was used as a supplementary source to identify difficult-to-access literature and recently published studies that may not have been indexed in the other databases. No restrictions were imposed on the publication year to ensure the most comprehensive coverage of the available scientific evidence.

The study selection process involved screening titles, abstracts, and full-text articles. Two reviewers independently performed the selection according to predefined inclusion and exclusion criteria. Any disagreements were resolved through discussion until a consensus was reached.

The extracted data were synthesised qualitatively and organised into major thematic categories corresponding to the objectives of this review. Particular emphasis was placed on identifying major research trends, commonly applied analytical techniques, and key knowledge gaps. Both recent publications and selected landmark studies that have substantially contributed to the current understanding of microplastics were included in the analysis.

Given the scoping nature of this review, no quantitative synthesis or formal methodological quality assessment of the included studies was performed. Instead, the findings are presented as a descriptive qualitative synthesis.

The literature search, study selection, and data extraction were performed independently by two reviewers using a standardised approach. This review was conducted and reported in accordance with the Preferred Reporting Items for Systematic Reviews and Meta-Analyses Extension for Scoping Reviews (PRISMA-ScR) guidelines. The completed PRISMA 2020 checklist is provided in the Supplementary Materials (Table S1), whereas the characteristics and key findings of all studies included in the scoping review are presented in Supplementary Materials (Table S2). The protocol for this scoping review was registered on the Open Science Framework (OSF; Center for Open Science) and is publicly accessible at https://osf.io/dpfbe/ (accessed on 29 July 2026).

## 9. Conclusions

Microplastics have become one of the most widespread environmental pollutants, being detected in surface waters, soils, the atmosphere, food products, and living organisms. They originate both from intentionally manufactured microscopic particles and from the secondary degradation of larger plastic debris. Their ubiquitous occurrence and capacity for long-range transport make microplastic pollution a truly global issue affecting virtually all ecosystems on Earth. Particular emphasis should be placed on adopting a life cycle perspective, which enables the identification of critical stages responsible for microplastic emissions and supports the development of effective mitigation strategies. Integrating life cycle assessment with advanced analytical methods and sustainable waste management practices will be essential for reducing microplastic pollution and minimising its environmental and human health impacts.

The development of advanced analytical techniques has substantially improved the identification and characterisation of microplastics. Microscopic, spectroscopic, and mass spectrometric methods enable detailed assessment of particle morphology, size, and chemical composition. Nevertheless, significant challenges remain in sample preparation, detection of the smallest particle fractions, and methodological standardisation. The harmonisation of analytical protocols remains one of the most important prerequisites for future progress in this field.

The evidence reviewed in this article indicates that microplastics may affect living organisms both directly and indirectly by transporting other environmental contaminants. Their capacity to adsorb heavy metals, pesticides, and persistent organic pollutants highlights their role as vectors of toxic substances. Furthermore, the detection of microplastics in human tissues, together with findings from experimental studies, suggests the possibility of adverse health effects, although the magnitude of these effects requires further investigation.

Mitigating microplastic pollution requires comprehensive strategies that include developing advanced filtration technologies, improving wastewater treatment, implementing bioremediation approaches, and implementing more efficient waste management systems. Preventive measures aimed at reducing plastic emissions at the source, in accordance with circular economy principles, are particularly important.

Although numerous studies have investigated microplastics, substantial research gaps remain, particularly concerning nanoplastics, bioaccumulation processes, atmospheric transport, and the long-term health effects of microplastic exposure. Future research should focus on developing standardised analytical methods, improving health risk assessment, and advancing innovative technologies to reduce microplastic emissions and environmental contamination.

Despite considerable progress, no single analytical technique currently provides a comprehensive characterisation of microplastics across all particle sizes and environmental matrices. Hybrid analytical workflows integrating spectroscopic and thermoanalytical techniques appear to represent the most promising direction for future methodological development.

Unlike previous reviews focusing primarily on analytical techniques or environmental occurrence, the present review integrates analytical approaches, environmental fate, life cycle assessment, and remediation strategies within a single comprehensive framework, providing a broader perspective for future research and environmental management.

## Figures and Tables

**Table 1:** Comparative assessment of analytical techniques used for micro- and nanoplastics characterisation.

Method	Particle Size Range	Polymer Identification	Morphological Analysis	Quantification	Suitability for Nanoplastics	Destructive	Automation Potential	Cost	LOD
Optical Microscopy	>20–50 µm	No	High	Limited	No	No	Moderate	Low	~20 µm
SEM	~1–2 µm	No	Very High	No	Limited	No	Low	Very High	~1 µm
SEM–EDX	~1–2 µm	Limited	Very High	No	Limited	No	Low	Very High	~1 µm
FTIR	20–5000 µm	High	Moderate	Limited	Limited	No	Moderate	High	~20 µm
μFTIR	10–20 µm	High	High	High	Moderate	No	High	Very High	10–20 µm
μRaman Spectroscopy	~1 µm	High	High	High	High	No	Moderate	Very High	~1 µm
Py-GC/MS	Whole sample	High	No	High	High	Yes	High	Very High	ng–µg
TED-GC/MS	Whole sample	High	No	High	High	Yes	High	Very High	ng–µg

Note: Qualitative ratings (Low, Moderate, High, Very High) were assigned based on a comparative evaluation of analytical capabilities, practical applicability, and methodological limitations reported in the reviewed literature. Abbreviations: FTIR—Fourier Transform Infrared Spectroscopy; SEM—Scanning Electron Microscopy; EDX—Energy Dispersive X-ray Spectroscopy; GC/MS—Gas Chromatography-Mass Spectrometry; LOD—Limit of Detection.

**Table 3:** Current knowledge, research challenges, knowledge gaps and future directions related to micro- and nanoplastics.

Research Area	Current State of Knowledge	Main Challenges	Research Gaps	Future Research Directions
Analytical methods	FTIR, μFTIR, Raman spectroscopy, SEM, Py-GC/MS and TED-GC/MS are the principal techniques for the identification and characterization of micro- and nanoplastics. Emerging approaches include NIR, NMR, XRF and AI-assisted image analysis.	Lack of standardized analytical protocols, limited comparability among studies, and difficulties in analyzing very small particles.	Reliable identification and quantitative determination of nanoplastics in complex environmental and biological matrices remain challenging.	Harmonization of analytical methods, automation of workflows, implementation of artificial intelligence, and development of more sensitive techniques for nanoplastic detection.
Environmental fate	Micro- and nanoplastics are present in aquatic, terrestrial, and atmospheric environments, as well as in food and living organisms. Their transport and transformation depend on particle properties and environmental conditions.	Complex transport, degradation, and transformation processes across environmental compartments.	Limited understanding of the long-term environmental fate and transformation of nanoplastics.	Long-term monitoring programmes, environmental modelling, and integrated studies across different environmental compartments.
Interactions with environmental contaminants	Micro- and nanoplastics adsorb heavy metals and persistent organic pollutants and may act as vectors for contaminant transport.	Assessment of adsorption and desorption processes under environmentally relevant conditions.	Insufficient knowledge regarding the combined effects of microplastics and co-occurring contaminants on organisms and ecosystems.	Multifactorial exposure studies under realistic environmental conditions and comprehensive environmental risk assessment.
Life Cycle Assessment	LCA is increasingly applied to evaluate the environmental impacts of plastics and microplastic emissions throughout the life cycle of plastic products.	Limited inventory data and insufficient integration of microplastic emissions into LCIA methodologies.	Lack of harmonized frameworks incorporating micro- and nanoplastics into LCA studies.	Development of standardized LCA methodologies and improved integration of microplastic emissions into environmental impact assessment.
Mitigation and remediation strategies	Current approaches include filtration technologies, wastewater treatment, bioremediation, recycling, and circular economy strategies.	Limited large-scale implementation and insufficient evidence regarding long-term effectiveness.	Limited knowledge on the long-term environmental sustainability and efficiency of remediation technologies.	Development of innovative removal technologies, preventive measures, circular economy solutions, and more effective regulatory frameworks.

## Data Availability

No new data were created or analyzed in this study. Data sharing is not applicable to this article.

## Supplementary Materials

The following supporting information can be downloaded at https://www.mdpi.com/article/10.3390/molecules31162789/s1. Table S1. Completed PRISMA 2020 Checklist. Table S2. Characteristics and key findings of studies included in the scoping review. Ref. [165] is cited in the Supplementary Materials.

## Abbreviations

The following abbreviations are used in this manuscript:

AI	Artificial Intelligence
BPA	Bisphenol A
CH_4_	Methane
CO_2_	Carbon Dioxide
EDX	Energy-Dispersive X-ray Spectroscopy
EFSA	European Food Safety Authority
FAO	Food and Agriculture Organization
FTIR	Fourier Transform Infrared Spectroscopy
GC/MS	Gas Chromatography–Mass Spectrometry
H_2_O_2_	Hydrogen Peroxide
IJERPH	International Journal of Environmental Research and Public Health
KOH	Potassium Hydroxide
LCA	Life Cycle Assessment
LCIA	Life Cycle Impact Assessment
LOD	Limit of Detection
MFs	Microfibers
MHETase	Mono(2-hydroxyethyl) terephthalic acid hydrolase
μFTIR (micro-FTIR)	Micro-Fourier Transform Infrared Spectroscopy
N_2_O	Nitrous Oxide
NaCl	Sodium Chloride
NaI	Sodium Iodide
NOAA	National Oceanic and Atmospheric Administration
PA	Polyamide
PAHs	Polycyclic Aromatic Hydrocarbons
PE	Polyethylene
PES	Polyester
PET	Polyethylene Terephthalate
PETase	Polyethylene Terephthalate Hydrolase
PHA	Polyhydroxyalkanoates
PLA	Polylactic Acid
POPs	Persistent Organic Pollutants
PP	Polypropylene
PRISMA-ScR	Preferred Reporting Items for Systematic Reviews and Meta-Analyses Extension for Scoping Reviews
PS	Polystyrene
PVC	Polyvinyl Chloride
Py-GC/MS (Py-GC-MS)	Pyrolysis–Gas Chromatography–Mass Spectrometry
Raman	Raman Spectroscopy
SAPEA	Science Advice for Policy by European Academies
SEM	Scanning Electron Microscopy
SEM-EDX	Scanning Electron Microscopy coupled with Energy-Dispersive X-ray Spectroscopy
TED-GC/MS	Thermal Extraction Desorption–Gas Chromatography–Mass Spectrometry
TRWPs	Tyre-and-Road Wear Particles
UV	Ultraviolet
WWTPs	Wastewater Treatment Plants
ZnCl_2_	Zinc Chloride
