# Supplementary material for: Micro- and Nanoplastics in the Environment: Analytical Approaches, Environmental Fate, Life Cycle, and Remediation Strategies—A Scoping Review
Source: Molecules. 2026 Aug 11;31(16):2789. doi: 10.3390/molecules31162789 (PMC13515896; doi:10.3390/molecules31162789)
Supplement: Supplementary file 1 [file molecules-31-02789-s001.zip › Supplementary Materials (Table S2).pdf]

Supplementary Materials (Table S2). Characteristics and key findings of studies included in the scoping review

Author	Year	Study type	Main topic	Key findings	Reference
Thompson et al.	2024	Review	Twenty years of microplastic pollution research	Summarised two decades of microplastic research, highlighting sources, environmental distribution, biological effects, human exposure, and the need for effective mitigation strategies.	[1]
Frias & Nash	2019	Review	Definition of microplastics	Proposed an expanded definition of microplastics that incorporates particle size, origin, and physicochemical properties to improve consistency in research and legislation.	[2]
Gigault et al.	2018	Review	Definition of nanoplastics	Proposed a definition of nanoplastics as unintentionally produced particles ranging from 1 to 1000 nm exhibiting colloidal behaviour.	[3]
Plastics Europe	2024	Report	Global plastics production and waste	Presents updated statistics on global and European plastics production, demand, and waste management.	[4]
Barnes et al.	2009	Review	Global accumulation and fragmentation of plastic debris	Reviewed the global distribution, persistence, fragmentation, and ecological impacts of plastic debris, highlighting the increasing abundance of microplastics.	[5]
Fendall & Sewell	2009	Original research	Microplastics in facial cleansers	Demonstrated that facial cleansers contain polyethylene microbeads capable of entering marine ecosystems through wastewater.	[6]
Cole et al.	2011	Review	Microplastics in the marine environment	Reviewed sources, distribution, analytical methods, and ecological impacts of marine microplastics while identifying priorities for future research.	[7]
Wright & Kelly	2017	Review	Microplastics and human health	Reviewed potential human health effects of microplastic exposure, emphasising particle toxicity, chemical contaminants, and the need for exposure assessment.	[8]
Browne et al.	2011	Original research	Sources of shoreline microplastics	Identified wastewater-derived synthetic textile fibres as a major source of microplastic contamination on shorelines worldwide.	[9]
Napper & Thompson	2016	Original research	Release of fibres during	Showed that washing synthetic textiles releases hundreds of thousands of microplastic fibres, with fibre release	[10]

			domestic washing	depending on fabric type and washing conditions.	
De Falco et al.	2018	Original research	Microplastic release during textile washing	Demonstrated that synthetic textile washing releases large quantities of microfibres, with release depending on fabric type and washing conditions.	[11]
Kole et al.	2017	Review	Tyre wear as a source of microplastics	Identified tyre wear as a major and underestimated source of environmental microplastics, contributing to aquatic, terrestrial, and airborne pollution.	[12]
Jambeck et al.	2015	Original research	Plastic waste entering oceans	Estimated global inputs of mismanaged plastic waste from land into the oceans and highlighted the growing contribution of coastal populations to marine plastic pollution.	[13]
Andrady	2017	Review	Plastic materials in microplastics	Reviewed the physicochemical properties, degradation processes, and environmental behaviour of plastics forming microplastics.	[14]
Andrady	2011	Review	Microplastics in the marine environment	Summarised the occurrence, sources, transport, degradation, and ecological impacts of marine microplastics.	[15]
Li et al.	2018	Review	Microplastics in freshwater systems	Reviewed the occurrence, environmental effects, and analytical methods used for detecting microplastics in freshwater ecosystems.	[16]
Danopoulos et al.	2020	Systematic review	Microplastics in drinking water	Systematically reviewed the occurrence of microplastics in drinking water and highlighted the need for harmonised analytical methodologies.	[17]
Corradini et al.	2019	Original research	Microplastics in agricultural soils	Demonstrated the accumulation of microplastics in agricultural soils following repeated sewage sludge application.	[18]
Allen et al.	2019	Original research	Atmospheric transport of microplastics	Provided evidence that atmospheric transport can deposit microplastics in remote mountain environments.	[19]
Bergmann et al.	2019	Original research	Microplastics in snow	Demonstrated widespread contamination of snow from the Alps to the Arctic, indicating long-range atmospheric transport of microplastics.	[20]
Cox et al.	2019	Original research	Human dietary intake of	Estimated annual human intake of microplastics from food, beverages and air.	[21]

			microplastics		
ISO	2023	Report	Microplastic terminology	Established standardized terminology and size classification for plastic particles.	[22]
Boucher & Friot	2017	Report	Primary microplastic sources	Identified and quantified the main global sources of primary microplastics.	[23]
Rochman & Hoellein	2020	Review	Global plastic pollution	Summarized the global distribution and environmental transport of plastic pollution.	[24]
Bank & Hansson	2022	Review	Microplastic cycle	Presented the concept of the microplastic cycle across environmental compartments.	[25]
EFSA Panel on Contaminants in the Food Chain	2021	Report	Microplastics in food	Assessed current knowledge on micro- and nanoplastics in food, especially seafood, and highlighted major data gaps.	[26]
Danopoulos et al.	2022	Systematic review	Microplastics in food and beverages	Reviewed and quantified microplastic contamination in food and beverages using meta-regression.	[27]
Prata	2018	Review	Airborne microplastics	Reviewed sources, exposure routes and potential health effects of airborne microplastics.	[28]
Leslie et al.	2022	Original research	Microplastics in human blood	Demonstrated and quantified microplastic particles in human blood samples.	[29]
Jenner et al.	2022	Original research	Microplastics in human lung tissue	Detected microplastics in human lung tissue using µFTIR spectroscopy.	[30]
Ragusa et al.	2021	Original research	Microplastics in human placenta	Demonstrated the presence of microplastics in human placental tissue.	[31]
Ragusa et al.	2022	Original research	Microplastics in human breast milk	Detected microplastics in human breast milk samples.	[32]
Vethaak & Legler	2021	Review	Microplastics and human health	Reviewed current evidence on human exposure and potential health effects of microplastics.	[33]
Campanale et al.	2020	Review	Human health effects of microplastics	Summarized potential toxicological effects of microplastics and plastic additives on human health.	[34]
Hirt & Body-Malapel	2020	Review	Immunotoxicity of	Reviewed intestinal and immunological effects associated with microplastic exposure.	[35]

			microplastics		
Rochman et al.	2013	Original research	Sorption of organic contaminants	Demonstrated long-term sorption of organic contaminants onto different plastic polymers.	[36]
Wang et al.	2017	Review	Microplastics in soils	Reviewed sources, occurrence and environmental impacts of microplastics in soils.	[37]
Teuten et al.	2009	Review	Transport of chemicals by plastics	Reviewed the transport and release of chemicals from plastics to organisms and the environment.	[38]
SAPEA	2019	Report	Microplastics in nature and society	Summarized current scientific evidence on microplastics and identified key research needs.	[39]
Hidalgo-Ruz et al.	2012	Review	Identification methods	Reviewed methods for the identification and quantification of environmental microplastics.	[40]
Primpke et al.	2018	Original research	FTIR reference database	Developed an FTIR reference database for automated microplastic identification.	[41]
Käppler et al.	2016	Review	Vibrational microspectroscopy	Reviewed FTIR and Raman microspectroscopy for environmental microplastic analysis.	[42]
Shim et al.	2018	Review	Marine microplastics	Summarized the abundance, distribution and composition of marine microplastics.	[43]
Löder & Gerdts	2015	Review	Detection methodology	Reviewed analytical methodologies used for microplastic detection and identification.	[44]
Song et al.	2015	Original research	Comparison of analytical methods	Compared microscopic and spectroscopic methods for microplastic identification.	[45]
Löder et al.	2015	Original research	Micro-FTIR imaging	Developed an FPA-based micro-FTIR imaging method for microplastic analysis.	[46]
Masura et al.	2015	Report	Laboratory methodology	Provided standardized laboratory protocols for quantifying microplastics in water and sediments.	[47]
Norén	2007	Report	Coastal microplastics	Documented the occurrence of small plastic particles in Swedish coastal waters.	[48]
Beckingham et al.	2023	Review	Hot needle test	Evaluated the reliability of the hot needle test for microplastic identification.	[49]
Fries et al.	2013	Original research	Py-GC/MS identification	Demonstrated polymer and additive identification using Py-GC/MS and SEM.	[50]
Goldstein et al.	2003	Book	Scanning electron microscopy	Presented the principles and applications of SEM and X-ray microanalysis.	[51]

Fotopoulou & Karapanagioti	2012	Original research	Plastic pellet surface properties	Characterized the surface properties of weathered plastic pellets.	[52]
Zhang et al.	2015	Original research	Floating microplastics	Demonstrated the accumulation of floating microplastics behind the Three Gorges Dam.	[53]
Verla et al.	2019	Review	Microplastic–chemical interactions	Reviewed mechanisms and implications of microplastic interactions with toxic chemicals.	[54]
Goldstein et al.	2018	Book	Scanning electron microscopy	Presented advanced SEM and X-ray microanalysis techniques for material characterization.	[55]
Wang et al.	2020	Review	Toxicity to fish	Reviewed the bioavailability and toxic effects of microplastics in fish.	[56]
Liu et al.	2024	Original research	ATR-FTIR analysis	Developed a large-area ATR-FTIR method for detecting microplastics down to 1.3 µm.	[57]
Gicquel et al.	2024	Original research	FTIR spectra generation	Developed synthetic FTIR spectra to expand reference databases for automated microplastic identification.	[58]
Ramos & Dias	2025	Review	FTIR and Raman spectroscopy	Reviewed integrated FTIR and Raman spectroscopy for refined microplastic analysis.	[59]
Liu et al.	2024	Original research	Microplastics in human arteries	Detected microplastics in human arterial tissues using Py-GC/MS.	[60]
Zhao et al.	2023	Original research	Microplastics in human reproductive system	Detected microplastics in human testis and semen samples.	[61]
Mintenig et al.	2017	Original research	Wastewater analysis	Detected microplastics in wastewater treatment plant effluents using micro-FTIR imaging.	[62]
Cowger et al.	2020	Review	Image and spectral analysis	Reviewed image processing and spectral classification techniques used in microplastic research.	[63]
Hidalgo-Ruz et al.	2012	Review	Identification methods	Reviewed methods for the identification and quantification of environmental microplastics.	[40]
Sharma et al.	2022	Review	Microplastics in food and drinking water	Reviewed the occurrence and potential health risks of microplastics in food and drinking water.	[64]
van Emmerik et al.	2023	Original research	River monitoring	Proposed harmonized monitoring strategies for estimating riverine plastic pollution.	[65]
Caldwell et al.	2022	Review	Detection methods	Reviewed analytical methods for detecting micro-, submicron-, and nanoplastics.	[66]

Eriksen et al.	2013	Original research	Great Lakes pollution	Documented widespread microplastic pollution in the surface waters of the Laurentian Great Lakes.	[67]
Lippiatt et al.	2013	Report	Marine debris monitoring	Provided standardized protocols for marine debris monitoring and assessment.	[68]
Masura et al.	2015	Report	Laboratory methodology	Provided standardized laboratory protocols for quantifying microplastics in water and sediments.	[47]
Rillig	2012	Review	Microplastics in soils	Highlighted terrestrial ecosystems and soils as emerging environments affected by microplastic pollution.	[69]
Corradini et al.	2019	Original research	Agricultural soils	Demonstrated microplastic accumulation in agricultural soils following sewage sludge application.	[18]
EFSA Panel on Contaminants in the Food Chain	2021	Report	Microplastics in food	Evaluated current evidence on the occurrence of microplastics and nanoplastics in food.	[26]
Kosuth et al.	2018	Original research	Human exposure	Detected microplastics in tap water, beer, and sea salt.	[70]
Schwabl et al.	2019	Original research	Human exposure	Detected various microplastics in human stool samples.	[71]
Ibrahim et al.	2021	Original research	Human colon	Detected microplastics in human colectomy specimens.	[72]
Masura et al.	2015	Report	Laboratory methodology	Provided standardized laboratory methods for microplastic analysis in marine samples.	[47]
Rani et al.	2023	Review	Extraction methods	Reviewed extraction methods for isolating microplastics from complex environmental matrices.	[73]
Ershova & Frank	2025	Review	Separation techniques	Reviewed separation and purification methods for microplastics from environmental samples.	[74]
Larrea et al.	2025	Review	Extraction methods	Compared extraction methods used for different environmental matrices.	[75]
Song et al.	2015	Original research	Identification methods	Compared microscopic and spectroscopic techniques for microplastic identification.	[45]
Käppler et al.	2016	Review	Vibrational spectroscopy	Reviewed FTIR and Raman microspectroscopy for environmental microplastic analysis.	[36]
Claessens et al.	2013	Original research	Detection methods	Developed improved techniques for detecting microplastics in sediments and organisms.	[76]
Imhof et al.	2012	Original research	Sediment analysis	Developed an efficient density-separation method for sediment microplastics.	[77]
Coppock et al.	2017	Original research	Sediment extraction	Developed a portable method for extracting microplastics from marine sediments.	[78]

Karami et al.	2017	Original research	Fish analysis	Developed a high-performance protocol for extracting microplastics from fish tissues.	[79]
Cole et al.	2014	Original research	Biological samples	Developed a protocol for isolating microplastics from seawater and marine organisms.	[80]
Primpke et al.	2017	Original research	FTIR automation	Developed an automated FTIR microscopy workflow for microplastic analysis.	[81]
Araujo et al.	2018	Review	Raman spectroscopy	Reviewed Raman spectroscopy applications for microplastic identification.	[82]
Shim et al.	2018	Review	Marine microplastics	Reviewed the abundance, distribution, and composition of marine microplastics.	[43]
Cowger et al.	2021	Report	Spectral classification	Promoted open-source spectral databases for microplastic identification.	[83]
Turner	2017	Original research	Portable XRF	Demonstrated portable XRF for in situ elemental characterization of marine microplastics.	[84]
Soon et al.	2026	Original research	Raman + XRF	Combined Raman spectroscopy and XRF for identifying marine coating microplastics.	[85]
Bec et al.	2024	Original research	NIR spectroscopy	Evaluated portable NIR spectrometers for rapid on-site identification of soil microplastics.	[86]
Schmidt et al.	2025	Original research	NMR spectroscopy	Demonstrated NMR spectroscopy for quantifying polymer mixtures containing microplastics.	[87]
Hermsen et al.	2018	Review	Quality assessment	Proposed quality criteria for microplastic analysis in biota samples.	[88]
Prata et al.	2019	Review	Sampling methods	Reviewed methods for sampling and detecting microplastics in water and sediments.	[89]
Miller et al.	2017	Review	Sample recovery	Evaluated recovery methods for isolating microplastics from marine samples.	[90]
Fuller & Gautam	2016	Original research	Extraction method	Developed a pressurized fluid extraction method for microplastic quantification.	[91]
Cowger et al.	2020	Report	Reporting guidelines	Provided reporting guidelines to improve transparency and reproducibility in microplastic research.	[92]
Koelmans et al.	2019	Review	Freshwater and drinking water	Reviewed data quality and occurrence of microplastics in freshwaters and drinking water.	[93]
Teuten et al.	2009	Review	Chemical transport	Reviewed transport and release of plastic-associated chemicals into the environment and wildlife.	[38]
Rochman et al.	2013	Original research	Sorption of contaminants	Demonstrated long-term sorption of organic contaminants onto plastic pellets.	[36]

Wang et al.	2017	Review	Soil contamination	Reviewed the occurrence and impacts of microplastics in soils.	[37]
Fotopoulou & Karapanagioti	2012	Original research	Surface properties	Demonstrated that weathering alters surface properties of plastic pellets.	[52]
Bakir et al.	2014	Original research	POP sorption	Demonstrated competitive sorption of persistent organic pollutants onto microplastics.	[94]
Endo et al.	2013	Original research	Desorption kinetics	Investigated desorption kinetics of hydrophobic contaminants from plastic pellets.	[95]
Hartmann et al.	2019	Report	Definitions	Proposed a harmonized framework for defining and classifying plastic debris.	[96]
Koelmans et al.	2016	Review	Chemical vectors	Reviewed the role of microplastics as vectors of chemicals in aquatic environments.	[97]
Lithner et al.	2011	Original research	Polymer hazards	Ranked plastic polymers according to their environmental and human health hazards.	[98]
Campanale et al.	2020	Review	Human health	Reviewed potential health effects of microplastics and plastic additives.	[34]
Wright et al.	2013	Review	Marine organisms	Reviewed physical impacts of microplastics on marine organisms.	[99]
Lusher et al.	2017	Report	Fisheries	Summarized occurrence and impacts of microplastics in fisheries and aquaculture.	[100]
Farrell & Nelson	2013	Original research	Trophic transfer	Demonstrated trophic transfer of microplastics between marine organisms.	[101]
Vethaak & Legler	2021	Review	Human health	Discussed current evidence linking microplastics with human health risks.	[33]
Hirt & Body-Malapel	2020	Review	Immunotoxicity	Reviewed intestinal and immunological effects of microplastic exposure.	[35]
Rillig et al.	2019	Review	Soil ecosystems	Reviewed impacts of microplastics on plants and soil ecosystem functioning.	[102]
Li et al.	2023	Original research	Life Cycle Assessment	Linked micro- and nanoplastic sources with impacts on soil ecosystem services in LCA.	[103]
Askham et al.	2023	Original research	LCIA methodology	Proposed sampling and testing approaches supporting life cycle impact assessment of microplastics.	[104]
Landrigan et al.	2023	Report	Plastics and health	Summarized evidence linking plastics with human health and recommended policy actions.	[105]
Ullah et al.	2023	Review	Endocrine disruption	Reviewed endocrine-disrupting effects of micro- and nanoplastics in mammals.	[106]
Volsa et al.	2025	Systematic review & meta-analysis	Fertility	Synthesized evidence on effects of micro- and nanoplastics on mammalian fertility.	[107]

Cui et al.	2025	Review	Organ toxicity	Reviewed mechanisms of microplastic-induced organ damage involving macrophages.	[108]
Luzi et al.	2025	Review	Textile microfibers	Reviewed microfiber release, mitigation strategies and legislation.	[109]
Rødland et al.	2022	Original research	Tire wear particles	Characterized tire and road wear microplastic contamination in road tunnels.	[110]
Moita Neto & Silva	2023	Review	Sources of microplastics	Reviewed major environmental sources of microplastic generation.	[111]
Ramanayaka et al.	2024	Review	Agricultural soils	Reviewed fate of microplastics and additives in plastic mulch-amended soils.	[112]
Chandra & Walsh	2024	Review	Water contamination	Reviewed occurrence, fate and removal of microplastics in water.	[113]
Tumu et al.	2023	Review	Plastic recycling	Reviewed recycling systems and extended producer responsibility legislation.	[114]
Suzuki et al.	2022	Original research	Mechanical recycling	Demonstrated mechanical recycling as a source of microplastic emissions.	[115]
Quicker et al.	2022	Review	Chemical recycling	Reviewed opportunities and limitations of chemical plastic recycling.	[116]
Lett et al.	2021	Review	Cardiovascular health	Reviewed exposure routes and cardiovascular effects of micro- and nanoplastics.	[117]
Guo et al.	2022	Original research	Bottle recycling	Identified plastic bottle recycling as an overlooked source of microplastics.	[118]
Megha et al.	2025	Review	Human health	Reviewed environmental impacts and health hazards of microplastics.	[119]
Arif et al.	2024	Review	Environmental impacts	Reviewed sources, behavior, remediation and environmental impacts of micro- and nanoplastics.	[120]
An et al.	2026	Review	Environmental health	Reviewed impacts of microplastics on ecosystems and human health.	[121]
Xi et al.	2022	Review	Degradation	Reviewed environmental behavior and degradation methods of microplastics.	[122]
Osman et al.	2023	Review	Sources and remediation	Reviewed sources, toxicity and remediation strategies for microplastics.	[123]
Zhou et al.	2026	Original research	Sand filtration	Evaluated sustainable sand filtration for microplastic removal from irrigation water.	[124]
Wang et al.	2020	Original research	Biochar filtration	Demonstrated efficient removal of microplastics using biochar filtration.	[125]
LaRue et al.	2024	Original research	Flow cytometry	Developed a flow cytometry strategy for quantifying microplastics during membrane filtration.	[126]
Rhein et al.	2022	Original research	Sewage sludge	Introduced magnetic seeded filtration for efficient separation of microplastics from sewage sludge extracts.	[127]
Lee et al.	2023	Review	Microplastic removal	Reviewed physical and biomimetic methods for reducing microplastic accumulation.	[128]

Harley-Nyang et al.	2023	Review	Sewage sludge	Reviewed global occurrence, characteristics and distribution of microplastics in sewage sludge and biosolids.	[129]
Roy et al.	2025	Original research	Wastewater treatment	Evaluated removal, identification and characterization of microplastics in sewage treatment plants.	[130]
Nakao et al.	2021	Original research	Wastewater treatment	Investigated the circulation of microfiber pollution during sewage and sludge treatment.	[131]
Mahon et al.	2017	Original research	Sewage sludge	Assessed the influence of sludge treatment processes on microplastic abundance.	[132]
Chen et al.	2023	Original research	Sewage sludge	Characterized dissolved organic matter released from microplastics during hydrothermal sludge treatment and evaluated its toxicity.	[133]
Zhou et al.	2022	Review	Bioremediation	Reviewed microbial degradation and bioremediation strategies for micro- and nanoplastics.	[134]
Pacher-Deutsch et al.	2025	Review	Gut microbiota	Reviewed the role of bacteria in mitigating microplastic effects within the digestive system.	[135]
Savino et al.	2025	Review	Soil remediation	Reviewed the effects of micro- and nanoplastics on plant-assisted bioremediation.	[136]
Giosafatto & Porta	2026	Review	Bioplastics	Discussed tailored bioplastics as sustainable alternatives to conventional plastics.	[137]
Sintim et al.	2019	Original research	Biodegradable plastics	Demonstrated release of micro- and nanoparticles during in situ composting of biodegradable plastics.	[138]
Daghighi et al.	2023	Review	Soil fauna	Reviewed impacts of plastic contamination on terrestrial micro- and mesofauna and highlighted research gaps.	[139]
Hettiarachchi & Meegoda	2023	Review	Waste management policy	Reviewed policy measures needed to prevent microplastic pollution through improved waste management.	[140]
Schyns & Shaver	2021	Review	Mechanical recycling	Reviewed current technologies, challenges and opportunities in mechanical recycling of plastic packaging.	[141]
Awasthi et al.	2020	Review	Biotechnology	Discussed biotechnological approaches for managing microplastic waste.	[142]
Bajt	2021	Review	Environmental impacts	Reviewed the pathway from plastics to microplastics and their biological effects.	[143]
Long et al.	2022	Original research	Environmental history	Reconstructed historical microplastic deposition to reflect plastic production and waste management trends.	[144]
Kwiatkowska & Ormaniec	2024	Review	Wastewater microbiome	Reviewed microbial succession on microplastics in wastewater treatment plants.	[145]
Thompson et al.	2024	Review	Twenty years of	Summarized progress, challenges and future priorities of microplastics research.	[1]

			microplastic research		
Chartres et al.	2024	Systematic review	Human health effects	Evidence strongest for exposure; health effects still uncertain.	[146]
Amesho et al.	2023	Review	Waste management policies	Emphasized coordinated policies to reduce microplastic pollution.	[147]
Meizoso-Regueira et al.	2024	Modeling study	Agricultural soil contamination	Predicted continued accumulation of microplastics in soils.	[148]
Kaur & Chauhan	2024	Review	Biodegradable plastics	Bioplastics may also generate persistent microplastics.	[149]
Changlor et al.	2025	Experimental study	Biochar filtration	Biochar effectively retained microplastics depending on particle size and biofilm.	[150]
Zhao & Yang	2019	Review	Graphene nanocomposites	Overview of graphene-based materials; potential environmental applications.	[151]
Gaur et al.	2025	Review	Microplastic mitigation	Summarized emerging remediation technologies.	[152]
Du et al.	2021	Review	Degradation technologies	Compared physical, chemical and biological degradation mechanisms.	[153]
Debnath et al.	2024	Review	Impacts and mitigation	Comprehensive overview of impacts and mitigation strategies.	[154]
Pérez-Guevara et al.	2022	Review	Standardization	Highlighted underreporting and need for standardized methodologies.	[155]
Kadac-Czapska et al.	2023	Review	Microplastics in food	Reviewed definitions, sample preparation and analytical techniques.	[156]
Cverenkárová et al.	2021	Review	Food chain	Summarized occurrence and transfer of microplastics through the food chain.	[157]
Mári et al.	2021	Validation study	Freshwater sample preparation	Validated standardized protocol for freshwater samples.	[158]
Kedzierski et al.	2022	Review	Infrared spectroscopy	Biofouling may interfere with FTIR identification of microplastics.	[159]
Roslan et al.	2024	Scoping review	Human tissues	Summarized evidence of microplastics detected in human organs.	[160]
Bhatt & Chauhan	2023	Review	Freshwater ecosystems	Reviewed bioaccumulation, trophic transfer and biomagnification.	[161]
Dalvand & Hamidian	2023	Review	Wetlands	Summarized occurrence and distribution of microplastics in wetlands.	[162]
Luo et al.	2022	Review	Atmospheric	Review of analytical methods and atmospheric transport models.	[163]

			microplastics		
Zhou et al.	2021	Review	Trends in microplastic research	Bibliometric overview of global research development.	[164]
